# The Sequel to the Mansion House Meeting

**Published:** 1921-07-16

**Authors:** 


### THE SEQUEL TO THE MANSION HOUSE MEETING.

Although the meeting convened by King Edward's Hospital Fund on the 6th inst. was of a semi-private character, and representatives of the Press were not present, the report which has been issued by the Fund indicates that a most helpful discussion took place on the question of mass contributions, referred to by Lord Cave's Committee as likely to provide the key of the problem of hospital finance. Three forms of mass contribution were before the meeting—namely, organised weekly contributions of wage-earners on a large scale, and the two insurance schemes, known as the "Oxford" and the "Sussex," under which sums are given on a contractual basis. The "Sussex" scheme, in a modified form, has already been launched, as we recorded in last week's issue, by the London Hospital, St. Thomas's Hospital, and the Royal Free Hospital, acting in concert, who have formed an organising committee at 3 Fenchurch Avenue. The members of this provisional organisation committee are the Hon. Sir Arthur Stanley, Lord Dawson of Penn, Sir Alan G. Anderson, Sir Napier Burnett, Mr. W. McAdam Eccles, and Dr. J. F. Gordon Dill, acting as honorary secretary. It is interesting to note that Mr. McAdam Eccles has for some time given much consideration to the application to London of a provident scheme, and that the Metropolitan Counties Branch of the British Medical Association, of whose Council Mr. Eccles is a member, some little time ago drafted an amended form of the Sussex scheme, which was called "A National Provident Scheme for Hospital and Additional Medical Services," the title which is now adopted by the new Organising Committee.

From the discussion summarised in the report (see page 264), we gather that the points upon which there is division of opinion are: First, whether any large effort should be contractual as is an insurance scheme or purely benevolent as are the League of Mercy and the Hospital Saturday Fund. The second point is the question of areas. It is desirable in the opinion of some hospital leaders that each hospital, so far as London is concerned, should have its own area from which to gather patients, this being particularly desirable should the scheme take an insurance form. Another point which was raised is that if wage-earners are to be organised for the purpose of mass contribution they should have representation on the local committees as well as on the hospital committees. The only definite decision arrived at by the meeting was that the King's Fund should co-operate with the British Hospitals Association, the Hospital Sunday and Saturday Funds, and the League of Mercy in the organisation of local collections from employees throughout the London area. This attempt to arrange combined action is welcome, but at the moment the thoughts of many hospitals hesitating how best to proceed will probably centre on the office at 3 Fenchurch Avenue; for the discussion at the Mansion House showed that it is the practical details of organisation rather than the choice between the schemes which is troubling hospitals. We hope, therefore, that the organising committee will make public without delay the procedure that it recommends, lest it be overwhelmed with separate inquiries. The general attitude can perhaps be summarised as follows: The London, St. Thomas's, and the Royal Free Hospitals are trying the "Sussex" scheme, with its element of insurance; St. Bartholomew's Hospital is willing to try "Scheme A," organised weekly contributions without insurance, if it does not interfere with patients' payments; a scheme, by the way, in which the organisations of the League of Mercy and the Saturday Fund might help. The special hospitals, whose patients come from all parts, would presumably have to be affiliated to such a scheme because of the difficulty in their case of appealing to a definite area. It is, indeed, the area question which causes trouble because the Londoner lives in one place and works in another, and people often support a hospital from a distance.

At present all these details are being worked out by the Organising Committee, and the scheme is not finally complete, but we understand that an area or district limit is not contemplated, though there will at first be a number limit, and the income limit for an individual will be that of the National Health Insurance, *i.e.*, £250. In regard to the division of the proceeds, it will be recalled that under the "Sussex" scheme thirty-five-fortieths are divided between the co-operating hospitals, according to an estimate of the work performed; and of the sums paid to the hospitals 25 per cent. is placed in a fund at the disposal of the medical staff. Of the remaining five-fortieths one is reserved for the cost of laboratory work, one for secretarial work, one for *x*-ray work, and the rest to be divided between those hospitals which have had the heaviest demands. Since the "Sussex" scheme is limited to persons whose income does not exceed £260 per annum for a single member, £400 for a man and wife or a widow and one child, and £500 for a married couple and family, it is plain that other schemes can be organised with it. The "Sussex" scheme is designed to appeal to a certain section of the community, which a certain idea no less than a certain income characterises, as is shown by the fact that the subscribers (of £1, 30s., or £2, as the case may be), can pay it in one sum or in weekly contributions. These facts are not too familiar to bear repetition, and there is this reason for their restatement, that, while Mr. Cronshaw explained fully to Lord Cave's Commission how the Oxford scheme was started, the Sussex scheme is known only to experts. For these reasons the plan of campaign proposed for London by the Organising Committee of the National Provident Scheme is awaited with eager interest.

